# A Study Using a Monte Carlo Method of the Optimal Configuration of a Distribution Network in Terms of Power Loss Sensing

**DOI:** 10.3390/s110807823

**Published:** 2011-08-09

**Authors:** Hyun Ho Moon, Jong Joo Lee, Sang Yule Choi, Jae Sang Cha, Jang Mook Kang, Jong Tae Kim, Myong Chul Shin

**Affiliations:** 1 Department of Electronic and Electrical Engineering, Sungkyunkwan University, 300 CheonCheon-dong, Jangan-gu, Suwon, Gyeonggi-do 440-746, Korea; E-Mails: hhmoon@daelim.ac.kr (H.H.M); 2 Smart Grid Research Center, Korea Electrotechnology Research Institute, 665-4 Naeson-dong, Uiwang-si, Gyeonggi-do 4226-170, Korea; E-Mail: jongjoo@keri.re.kr; 3 Department of Mechatronics Engineering, Induk Institute of Technology, Wolgye 2-dong, Nowon-gu, Seoul 139-749, Korea; E-Mail: ppk99@induk.ac.kr; 4 Department of Media Engineering, Seoul National University of Science and Technology, 172 Gongreung 2-dong, Nowon-gu, Seoul 139-743, Korea; E-Mail: chajs@snut.ac.kr; 5 Electronic Commerce Research Institute, Dongguk University, 707 Seokjang-dong, Gyeongju, Gyeongsangbuk-do, 780-714, Korea; E-Mail: mooknc@gmail.com

**Keywords:** SmartGrid, distribution sensing, MicroGrid, distribution generator, optimal configuration, Monte Carlo

## Abstract

Recently there have been many studies of power systems with a focus on “New and Renewable Energy” as part of “New Growth Engine Industry” promoted by the Korean government. “New And Renewable Energy”—especially focused on wind energy, solar energy and fuel cells that will replace conventional fossil fuels—is a part of the Power-IT Sector which is the basis of the SmartGrid. A SmartGrid is a form of highly-efficient intelligent electricity network that allows interactivity (two-way communications) between suppliers and consumers by utilizing information technology in electricity production, transmission, distribution and consumption. The New and Renewable Energy Program has been driven with a goal to develop and spread through intensive studies, by public or private institutions, new and renewable energy which, unlike conventional systems, have been operated through connections with various kinds of distributed power generation systems. Considerable research on smart grids has been pursued in the United States and Europe. In the United States, a variety of research activities on the smart power grid have been conducted within EPRI’s IntelliGrid research program. The European Union (EU), which represents Europe’s Smart Grid policy, has focused on an expansion of distributed generation (decentralized generation) and power trade between countries with improved environmental protection. Thus, there is current emphasis on a need for studies that assesses the economic efficiency of such distributed generation systems. In this paper, based on the cost of distributed power generation capacity, calculations of the best profits obtainable were made by a Monte Carlo simulation. Monte Carlo simulations that rely on repeated random sampling to compute their results take into account the cost of electricity production, daily loads and the cost of sales and generate a result faster than mathematical computations. In addition, we have suggested the optimal design, which considers the distribution loss associated with power distribution systems focus on sensing aspect and distributed power generation.

## 1. Introduction

In contemporary society, power consumption is increasing and the types of power consumed in distribution systems are also being diversified. With this diversification of distribution systems, distributed generators used in existing systems can be subdivided into smaller units, called microgrids [1]. With the spread of such subdivided power systems, existing distribution systems will have a bilateral flow form rather than a unilateral flow, and they can be supplied with power from distributed generators other than from KEPCO (Korea Electric Power Corporation), and resell the unused power to KEPCO.

Upon announcing its vision and development strategy for the “New Growth Engine Industry” in January 2009, the Korean government announced the three major industrial areas and 17 fields selected as part of the New Growth Engine. Of the New Growth Engine Industries, a study related to “New and Renewable Energy” is regarded as the core of the Green Technology Industry [2]. Therefore studies concerning the Smart Grid, which is the next generation power grid system and sensing technology, are being conducted very widely due to increasing interest in New and Renewable Energy [3,4].

Such Distributed Generation systems using New and Renewable Energy and connected to Power Distribution Systems using sensing comprise the MicroGrid, a small-scale power system, which operates connected to the traditional grid. The application of smart grid technology has turned the MicroGrid into a system that can digitize in real-time all the processes of power generation, distribution and demand chain. For this reason, the MicroGrid connected to a Power Distribution System should be operated through an operation plan which guarantees economic efficiency, and the configuration of the Power Distribution System should be reorganized to accommodate this [5].

In this study, we evaluated an economic generating capacity for the MicroGrid based on the electricity generation cost and the resale value of the MicroGrid and the supply price of the main power source by the use of a Monte Carlo simulation as a probabilistic analysis. Then we calculated the distribution loss of the MicroGrid. Based on that result, we suggested the optimal configuration of the power distribution system that is able to increase the profit by reassignment of the power supply and utility sensing sectors.

## 2. Evaluation of the Economical Efficiency of the Distribution System

### 2.1. MicroGrid and SmartGrid

With the introduction of small-sized distributed generators and planned control, it is possible to control a power system network that can generate an amount of power large enough to be operable separately from the main power system network, and a part of a power system network with such capacity is called a Microgrid [5]. Small-sized power systems such as solar energy, wind power, fuel cells, micro turbines and combined heat and power systems are included in the MicroGrid. Of course, these installattions are also useful for sensing of the utility and network [6,7].

SmartGrid technology, which currently attracts as much attention as the MicroGrid, is the next generation intelligent electrical power system that will improve the reliability and efficiency of the power generation, distribution and transmission systems through the use of digital technology. Accordingly, the relationship between the power supplier and the end user becomes closer than the current relationship. The end user can consume the electricity during the time when an electric company provides-cheaper electricity and selects the time for the use of specific electrical appliances [8,9].

In order to operate such a MicroGrid and SmartGrid connected to a currently operating electrical power and sensing network, an evaluation of the economic aspects should be performed and it is also very important to establish a reliable standard for the evaluation.

### 2.2. Distribution Loss Rate

The distribution Loss Rate is the percentage expression for the degree of power loss which occurs through wire resistance and the likes whilst electricity generated from power plants is delivered to consumers through transmission lines, transformers and distribution lines [10]:

(1) $$\mathrm{Distribution}\;\mathrm{Loss}\;\mathrm{Rate}=\frac{\mathrm{the}\;\mathrm{amount}\;\mathrm{of}\;\mathrm{Distribution}\;\mathrm{power}\;-\;\mathrm{the}\;\mathrm{amount}\;\mathrm{of}\;\mathrm{power}\;\mathrm{sale}}{\mathrm{the}\;\mathrm{amount}\;\mathrm{of}\;\mathrm{Distribution}\;\mathrm{power}}\times 100\%$$

The distribution Loss is divided into two categories the loss from electrical characteristics and the loss from electric power management, and the detailed characteristics are shown in Table 1 below. The equation for calculating the power loss is given by [11]:

(2) $$3\;\mathrm{Phase}\;\;:\;\;W=3I_{m}^{2}\times H\times R_{e}\times T\times 10^{-3}(\mathrm{kWh})$$

(3) $$1\;\mathrm{Phase}\;\;:\;\;W=2I_{m}^{2}\times H\times R_{e}\times T\times 10^{-3}(\mathrm{kWh})$$

### 2.3. Monte Carlo Technique

The Monte Carlo technique, which is one of the best known simulation methods, is based on getting the probabilistic distribution of a number through statistics from repeatable experiments. This method is useful to find an approximate solutions using random numbers for problems that are hard or impossible to solve analytically or numerically using numeric equations or functions [12].

An advantage of the Monte Carlo technique is its ease of application. To get the accurate target value, an appropriate algorithm should be developed based on extensive background knowledge including various mathematical formulae. However, the Monte Carlo technique can give a relatively accurate value through simulation without such procedures. On the other hand, a disadvantage of the method is that the result is meaningless if the probability distribution of the input values and the range of mathematical modeling are not correct. Because the distribution of random numbers has a significant effect on the result of analysis, the random number generation function should be formulated properly according to the required range and distribution of random numbers.

**Figure 1.**
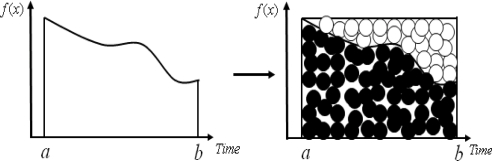
Monte Carlo integral calculus [12].

Figure 1 shows the Monte Carlo method which calculate approximate space using random number.

## 3. Simulation Data

### 3.1. Target System

The RBTS is a test system that consists of a total of six buses used to test the reliability of a transmission system. Among the six RBTS buses, the distribution line diagram and reliability indices for buses 2 and 4 are provided as power distribution systems gain more interest in terms of reliability. The RBTS Bus-2 system was employed for the test system and the simulation was applied to this test system. This test system was prepared for evaluating the power distribution system reliability and the peak load of overall system was 20 MW. In this paper, we completed the power distribution system by adding Distributed Generation to the RBTS Bus-2 system. Each peak load capacity used an offered value and the RBTS Bus-2 system was modified by dividing the overall system into four areas [13]. Each sector was categorized into a residential area, a business area, and a large scale industrial area.

The Distributed Generation power sources by area were wind power, solar energy and fuel cells, respectively, and each peak load and Distributed Generation power capacity by area is as shown in Table 2.

Data for the calculation of the distribution loss of the test system is shown in Table 3.

**Figure 2.**
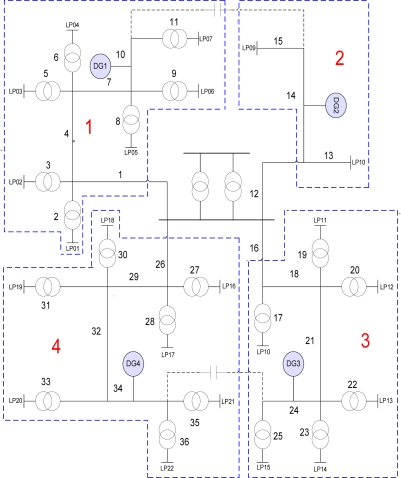
RBTS 2BUS System.

Figure 2 shows the RBTS 2 bus modifying Table 3.

### 3.2. Probability

The Monte Carlo simulation is a probabilistic method, and the reliability of its results is improved when objective data are used for its variables [15]. In this study, the probability distribution as in Table 4 was calculated based on the IEEE RTS daily load data, and the load was decided in proportion to the system to which the simulation was applied.

### 3.3. Factors for the Calculation of the Generation Cost of Distributed Generation

When the Monte Carlo simulation was performed, in order to calculate the Generating Capacity which provides the largest profit for the amount of power sales, the generation cost by Distributed Generation and probability distribution were used as simulation data. In order to adopt objective data, Table 5 and Table 6 utilized the indices of the KEPCO (Korea Electric Power Corporation) and the KEMCO (Korea Energy Management Corporation), respectively.

### 3.4. Distribution Loss Rate

The distribution loss rates by a line of the test system, which were calculated by considering the line data and load data provided by the RBTS Bus-2 system, are shown in Table 7 below. Distribution loss rates in this paper were calculated by applying an equal loss factor and the equivalent resistance of the line, and were computed in proportion to the line length and load capacity.

## 4. Optimal Configuration Simulation

### 4.1. Monte Carlo Simulation

In order to calculate the generation capacity that maximizes the economic profit in each distributed generator, a Monte Carlo simulation was performed by generating random numbers and calculating the probability distribution using a representative Excel spreadsheet and by applying various simulation data mentioned in the previous session. The input data of the simulation were the generation cost and sales cost calculated from the data of the Korea Electric Power Corporation and the Korea Energy Management Corporation, and the probability distribution according to the capacity obtained from the daily load. The simulation was performed five times repeatedly using 5000 random numbers, and the generation of the maximum profit was calculated using the mean values.

**Figure 3.**
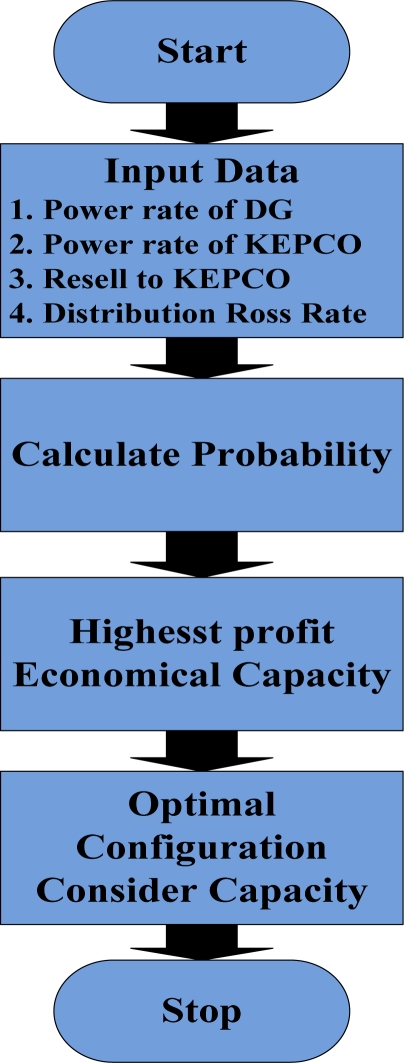
Flow chart of Monte Carlo Simulation.

The number of random numbers, which influences the results of the simulation significantly, was determined through repeated experiments. When the simulation was performed with fewer random numbers, the variation of the generating capacity required to gain the maximum profit was very large, whilst the variation of the generating capacity decreased and a more accurate result was derived as the number of random numbers was increased. However the accuracies tended to converge with increasing numbers of random numbers, and when the number of random numbers was over 5000, the same result was obtained. Because the use of a lot of random numbers extends the simulation time, five thousand random numbers were employed in this study in order to minimize the simulation time required to achieve the desired accuracy. Figure 3 is a flowchart of the Monte Carlo simulation used in this paper.

### 4.2. Profit Graph of DG

**Figure 4.**
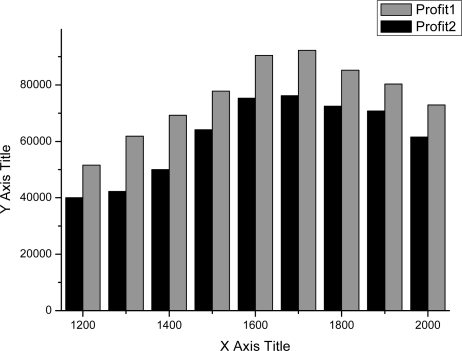
Profit graph of DG1 (Wind).

**Figure 5.**
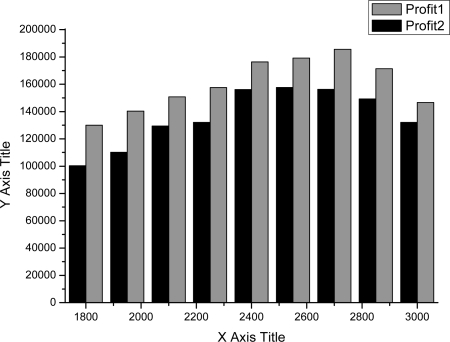
Profit graph of DG2 (Wind).

**Figure 6.**
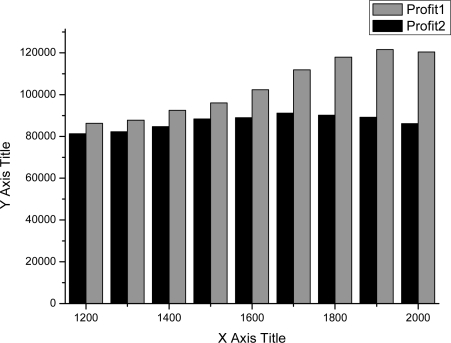
Profit graph of DG3 (PV).

**Figure 7.**
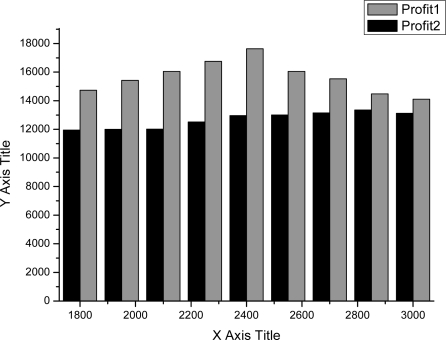
Profit graph of DG4 (Fuel Cell).

As shown in Figures 4–7, we can find the generating capacity which provided the largest profit through distributed power generation based on the simulation results. These figures present a comparison between two maximum profit amounts (profit1 and profit2) which were calculated before and after applying the distribution loss rate, respectively.

### 4.3. Optimal Configuration

**Figure 8.**
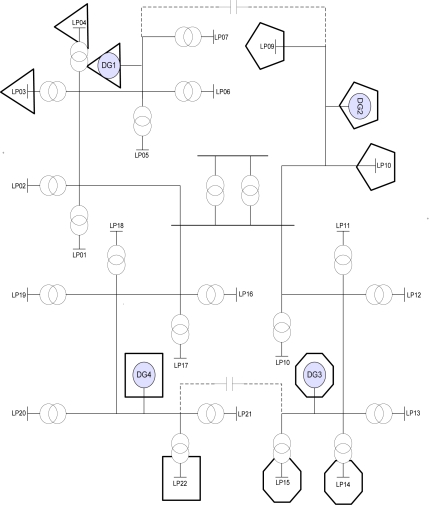
Optimal configuration of RBTS 2BUS system.

By comparing the maximum profits obtained through simulation with the value calculated as a function of the distribution loss, the generating capacity which was able to produce the maximum profit from distributed power generation was determined. With these results, we can estimate the generating capacity of a distribution system which can achieve the most economically efficient operation. Figure 8 shows the optimal electricity supply area of the distribution system reorganized using these values.

## 5. Conclusions

This study suggests the optimal configuration of a distribution system by evaluating the economical efficiency of the MicroGrid, a small-scale power system, which can be operated independently with distributed generation connected to a distribution system and sensing. The optimal configuration was achieved by the use of a Monte Carlo simulation which can offer an approximate value more rapidly than numerical methods. We derived the economical indices from the sum of the generating capacity for the maximum profit and the distribution loss rate through the simulation. According to these indices, the distribution system achieved the most economically efficient configuration through the alteration of the existing generating capacity and supply area. The evaluation of the economic efficiency of a distribution system is a necessary process and a Monte Carlo simulation, which can offer an approximate result value rapidly by a simple approach, is believed to be a very useful tool in the numerically complicated evaluation of economic efficiency. We believe that a prospective study can be conducted through the study of the probability distribution values of various detailed indices of a distribution system and the cost variation of generating electricity depending on the RTP (Real Time Price).

## Figures and Tables

**Table 1.** Distribution Loss detail characteristics.

**Loss from Electrical Characteristics**	**Loss from Electric Power Management**
- resistance loss of the overhead lines	
- corona loss	- conduction losses
- load loss of transformer resistance loss of underground lines	- loss increase by customer power factor decrease

- dielectric loss	- meter errors
- loss from low-voltage line and incoming line	
- loss from electric meter	

**Table 2.** Peak capacity of the area and capacity of DG [14].

**Areas**	**Capacity[kW]**	**Capacity of DG[kW]**
1 (Residential)	5933.8	2000 (Wind)
2 (Industrial)	3500	3000 (Wind)
3 (Residential)	5046.1	2000 (PV)
4 (Business )	5520.7	3000 (Fuel Cell)

**Table 3.** Loss data [14].

**Line Length(km)**	**Line Number**
0.60	2, 6, 10, 14, 17, 21, 25, 28, 30, 34
0.75	1, 4, 7, 9, 12, 16, 19, 22, 24, 27, 29, 32, 35
0.80	3, 5, 8, 11, 13, 15, 18, 20, 23, 26, 31, 33, 36

**Table 4.** Peak capacity of area and capacity of DG.

**Ratio**	**Operating Time [Hour]**	**Probability [%]**	**Accumulated Probability [%]**
0.60	4	0.17	0.00
0.65	2	0.08	0.17
0.70	1	0.04	0.25
0.75	2	0.08	0.29
0.80	0	0	0.38
0.85	1	0.04	0.38
0.90	1	0.04	0.42
0.95	7	0.29	0.46
1 (Peak)	6	0.25	0.75

**Table 5.** The power rate of KEPCO in 2008 [16].

**Classification**	**Power Rate**
Residential [Won/kWh]	55.10
Commercial [Won/kWh]	67.90
Industrial [Won/kWh]	65.80

**Table 6.** The power rate of DG [16].

**Classification**	**Operating Time [Hour]**
Wind Turbine Generator [Won/kWh]	107.29
Solar Power Generator [Won/kWh]	667.38
Fuel Cell [Won/kWh]	282.54

**Table 7.** The power rate of DG.

**Ratio**	**Line Number**	**Distribution Loss Probability**
0.30	34	0.01
0.35	10, 28	0.02
0.40	25, 30	0.03
0.45	6, 14	0.03
0.50	7, 26, 35	0.08
0.55	2, 27	0.05
0.60	17, 24, 36	0.09
0.65	1, 21, 22	0.09
0.70	4, 20, 32	0.11
0.75	9, 21, 19	0.10
0.80	5, 11, 15, 16	0.13
0.85	3, 8, 12, 33	0.01
0.90	13, 18	0.06
0.95	23, 29	0.07
1 (Peak)	31	0.03
